# Development, adaptation, and validation of a cognitive assessment battery harmonized with the National Alzheimer's Coordinating Centre's (NACC) Uniform Data Set (UDS) in Botswana

**DOI:** 10.1002/alz70858_106001

**Published:** 2025-12-26

**Authors:** Lingani Mbakile‐Mahlanza

**Affiliations:** ^1^ Global Brain Health Institute, San Francisco, CA, USA; University of Botswana, Gaborone, Botswana

## Abstract

**Background:**

The world's population is rapidly aging accompanied by a drastic rise in the prevalence and incidence of Alzheimer's disease and related dementias (ADRD), especially in low‐ and middle‐income countries (LMICs). It is estimated that 75% of people with dementia are not diagnosed globally, with underdiagnosis rates as high as 90% in LMICs. Lack of appropriate neuropsychological assessment instruments, their variable implementation, and limited local expertise are major contributors to these disparities. To address these gaps, we report on the design and methodology of a cognitive assessment protocol harmonized with the NACC UDS and the Multi‐Partner Consortium to Expand Dementia Research in Latin America (ReDLat) in Botswana.

**Method:**

We used the International Test Commission guidelines to translate and culturally adapt neuropsychological measures from the NACC UDS and ReDLat batteries. Qualitative methods, including focus groups and expert interviews, were used to analyse the proposed measures for cultural applicability of test stimuli, constructs, and instructions. Pilot data on the face and content validity of the adapted battery was collected in a small sample with varying age, educational level, and socioeconomic status characteristics.

**Result:**

Qualitative findings indicated standardising test instructions was challenging particularly for speeded tasks. Tasks using clock drawing (MoCA) and set‐shifting (Trailmaking Test B) paradigms were deemed inappropriate or have poor validity in Batswana adults with lower education. The expert panel reached a consensus that verbal stimuli (e.g., names in Craft Story Test, items on the Multilingual Naming Test) were not appropriate to local context and needed to be revised with more appropriate content. We are currently administering the adapted battery and aim to report expanded quantitative findings in 40 neurologically healthy older adults by July 2025.

**Conclusion:**

The study is the first to culturally adapt and validate cognitive assessments in Botswana. Our qualitative findings provide valuable cultural, social, and linguistic insights into considerations for test selection and adaptation procedures for ADRD studies in Africa and can be informative for other Global South settings. We will additionally report the quantitative findings including psychometric properties of the adapted battery and examination of the associations of cognitive scores with key demographic variables.

